# Association between visceral adiposity index and cancer risk in the UK Biobank cohort

**DOI:** 10.1002/cncr.35576

**Published:** 2024-10-03

**Authors:** Solange Parra‐Soto, Jirapitcha Boonpor, Nathan Lynskey, Carolina Araya, Frederick Ho, Jill P. Pell, Carlos Celis‐Morales

**Affiliations:** ^1^ Department of Nutrition and Public Health, Faculty of Health and Food Science Universidad del Bío‐Bío Chillan Chile; ^2^ School of Health and Wellbeing University of Glasgow Glasgow UK; ^3^ School of Cardiovascular and Metabolic Health University of Glasgow Glasgow UK; ^4^ Faculty of Public Health Chalermphrakiat Sakon Nakhon Province Campus Kasetsart University Sakon Nakhon Thailand; ^5^ Education, Physical Activity, and Health Research Unit Human Performance Lab University Católica del Maule Talca Chile

**Keywords:** cancer, cohort, obesity, visceral adiposity index, visceral fat

## Abstract

**Background:**

The visceral adiposity index (VAI) is a marker of visceral fat accumulation and metabolic dysfunction, but there is limited evidence of its association with cancer. The objective of this study was to investigate associations between the VAI and both incident cancer at 23 sites and all‐cause cancer.

**Methods:**

In total, 385,477 participants (53.3% women; mean age, 56.3 years) from the UK Biobank prospective cohort were included in this study. The median follow‐up was 8.2 years (interquartile range, 7.3–8.9 years). The VAI was calculated using formula the published by Amato et al. and was categorized into sex‐specific tertiles. Twenty‐four incident cancers were the outcomes. Cox proportional hazard models were adjusted for sociodemographics, lifestyle factors, and multimorbidity counts.

**Results:**

Over the follow‐up period, 47,882 individuals developed cancer. In the fully adjusted models, the VAI was associated with a higher risk of six cancer sites. Individuals in the highest tertile, compared with those in the lowest tertile, had higher risks of uterine (hazard ratio [HR], 2.09; 95% confidence interval [CI], 1.76–2.49), gallbladder (HR, 1.83; 95% CI, 1.26–2.66), kidney (HR, 1.39; 95% CI, 1.18–1.64), liver (HR, 1.25; 95% CI, 1.00–1.56), colorectal (HR, 1.14; 95% CI, 1.05–1.24), and breast (HR, 1.11; 95% CI, 1.03–1.19) cancers and of all‐cause cancer (HR, 1.05). There was no evidence of a nonlinear association between the VAI and cancer risk.

**Conclusions:**

The VAI was associated with six cancer sites and with all‐cause cancer. The prognostic and etiologic roles of visceral fat accumulation and dysfunction in cancer warrant further research.

## Abstract 2

The objective of this study was to determine the association between the visceral adiposity index and both incident cancer across 23 sites and overall cancer risk. By using data from 385,477 participants in the UK Biobank cohort, the results indicated that individuals in the highest visceral adiposity index tertile had a significantly elevated risk of uterine, gallbladder, kidney, liver, colorectal, and breast.

## INTRODUCTION

There were 19.3 million new cancer cases in 2020^1^ and, by 2040, this number is expected to increase to 27.5 million.^2^ Cancer is also the leading cause of death in the world.^1^ Obesity has a strong association with increased incidence and premature mortality from some types of cancer.^3^, ^4^, ^5^, ^6^ In 2018, the World Cancer Research Fund reported that high body mass index (BMI) is associated with higher risk of 12 cancers, including colorectal, postmenopausal breast, esophageal, pancreatic, liver, kidney, oral, pharyngeal and laryngeal, stomach cardia, gallbladder, ovarian, (advanced) prostate, and uterine cancers.^7^ The World Cancer Research Fund report also highlighted the lack of evidence regarding the association of cancer with other markers of adiposity.^7^

Visceral fat accumulation, characterized by the extensive internal deposition of fat around abdominal organs, is a key factor in the development of certain cancers.^6^, ^8^, ^9^ The visceral adiposity index (VAI) is suggested as an effective indicator of such accumulation and has been associated with metabolic and hormonal disturbances.^10^ These disturbances can lead to a proinflammatory state and oxidative stress, thereby creating conditions that may promote the onset of cancer.^6^, ^8^, ^9^ The VAI integrates anthropometric measurement (waist circumference [WC] and BMI) and metabolic parameters (including triglyceride [TG] levels and high‐density lipoprotein [HDL] cholesterol), offering a comprehensive assessment of visceral adiposity and metabolic health.^10^ Evidence indicates that the VAI is associated with inflammatory markers, such as cytokines (which are related to chronic inflammation), that have been linked to the development of cancer and other diseases.^11^, ^12^, ^13^ It has been demonstrated that the VAI is a useful marker in polycystic ovary syndrome,^14^, ^15^ with potential use as a screening tool.^16^ In addition, the VAI reportedly was more sensitive for the evaluation of total body fat and fat accumulation in the central abdominal region.^17^

The VAI has known associations with type 2 diabetes,^12^ chronic kidney disease,^13^ cardiovascular diseases,^14^ and increased risk of all‐cause mortality.^15^, ^16^ However, the association with cancer risk has been limited to small studies examining breast cancer^17^ and colorectal cancer (CRC).^18^ The only study available that investigated the link between the VAI and CRC had a retrospective design, included 27,921 participants, had a short follow‐up duration (median, 4.4 years), and did not investigate specific colon sites.^18^ Similarly, the evidence for breast cancer comes from a single, small, cross‐sectional study, which included 116 cases and 226 controls and indicated that individuals with breast cancer had higher VAI levels.^17^ Furthermore, no previous studies have investigated associations with other cancer sites or whether the VAI would have better predictive ability for detecting cancer risk than traditional markers of adiposity, such as the BMI. There is a potential causal mechanistic link between the VAI and cancer because the VAI is associated with inflammatory markers, such as cytokines, which are related to chronic inflammation. Chronic inflammation, in turn, has been linked to the development of cancer and other diseases.^19^, ^20^, ^21^

Investigating the role of the VAI in cancer risk assessment is essential to determine its predictive value and whether it could surpass the predictive ability of traditional adiposity markers, such as the BMI, which are simple and cost‐effective in clinical settings. By elucidating the associations between the VAI and incident cancers, the objective of the current study was to provide insights into whether the VAI could be a better tool for early cancer risk identification. Therefore, in this prospective study, we investigated the associations between the VAI and incident cancers at 23 disease sites, as well as all‐cause cancer, accounting for potential nonlinear associations.

## MATERIALS AND METHODS

### Study design

UK Biobank recruited greater than 500,000 participants (aged between 37 and 73 years; 56.3% women) between 2006 and 2010.^24^ Participants attended one of 22 assessment centers across England, Scotland, and Wales, where they completed a self‐administered, touch‐screen questionnaire, a face‐to‐face interview, and provided biologic samples. Eligibility criteria for participation included being registered with the National Health Service and living within a reasonable distance of one of the assessment centers.^25^, ^26^ The outcomes used in this study were incident cancers at 23 sites, including four subgroups for CRC, as well as all cancers combined. Of the 23 cancers, 17 were relevant to both men and women, two were specific to men (testicular and prostate), and four were specific to women (breast, uterine, cervical, and ovarian).

### Participant follow‐up

Date of death was obtained from death certificates held by the National Health Service Information Center (England and Wales) and the National Health Service Central Register Scotland (Scotland). Date and cause of cancer were obtained through record linkage to National Health Service Digital (England and Wales) and Scottish Morbidity Records (Scotland). Detailed information about the linkage procedures can be found at http://content.digital.nhs.uk/services, July 15, 2024. At the time of analysis, mortality data were available up to February 28, 2021. Therefore, mortality analyses were censored at this date or on the date of death, whichever occurred first. Cancer registry information data were available until October 31, 2015, for Scotland and until July 31, 2019, for England and Wales, resulting in analyses of incident outcomes being censored at these dates or at the date of relevant hospitalization or death, whichever occurred first. We defined incident cancer as fatal or nonfatal events. International Classification of Diseases, 10th revision (ICD‐10) codes were used to define the following 23 cancers sites, including four subgroups for CRC: all‐cause cancer (C00–C97, excluding nonmelanoma skin cancer [C44]), head and neck cancer (C00–C14), esophageal cancer (C15), stomach cancer (C16), CRC (C18, C19, and C20), colon cancer (C18.0), proximal colon cancer (C18.0–C18.4), distal colon cancer (C18.5, C18.7), rectal cancer (C19–C20), liver cancer (C22), gallbladder cancer (C23–C24), pancreatic cancer (C25), lung cancer (C33–C34), malignant melanoma (C43), breast cancer (C50), uterine cancer (C54–C55), cervical cancer (C53), ovarian cancer (C56), prostate cancer (C61), testis cancer (C62), kidney cancer (C64–C65), bladder cancer (C67), brain cancer (C70–C72), thyroid cancer (C73), Hodgkin lymphoma (C81), non‐Hodgkin lymphoma (C82–C86, C96), multiple myeloma (C88–C90), and leukemia (C91–C95).

### Exposure

Anthropometric measurements, including body weight, height, and WC, were collected at baseline by trained staff using standardized protocols.^27^ Height was measured to the nearest centimeter using a Seca 202 stadiometer, and body weight was measured to the nearest 0.1 kg using a Tanita BC‐418 body composition analyzer. BMI was calculated as weight (kg) divided by height (m) squared.^28^ WC was measured at the natural indent (or umbilicus if the natural indent could not be observed). Serum C‐reactive protein (CRP) was measured by immunoturbidimetric high‐sensitivity analysis on a Beckman Coulter AU5800 chemistry analyzer and reported as mg/L.^29^ TG and HDL cholesterol levels were analyzed from serum and packed red blood cell samples. The VAI was calculated using the following formulae: men, VAI = (WC/[39.68 ± 1.88 × BMI]) × (TG/1.03) × (1.31/HDL); women, VAI = (WC/[36.58 ± 1.89 × BMI]) × (TG/0.81) × (1.52/HDL).^10^ Because distribution of the VAI differs by sex, the VAI was then categorized into sex‐specific tertiles, as presented in Table S1.

### Covariates

Age, sex, ethnicity, income, smoking status, diet (intake of fruits and vegetables, red and processed meat, and oily fish), alcohol intake (ordinal variable: daily, two to four times a week, once or twice a week, from one to three times a month, special occasions or never), and health‐related variables were self‐reported at the baseline assessment.^27^ Participants provided their date of birth, which was used to calculate their age at the time of enrolment in the study. Participants indicated their biologic sex as male or female. Participants reported their ethnic background based on predefined categories provided in the questionnaire, which included options such as White, Black or Black British, Asian or Asian British, mixed, and other ethnic group.^27^ The Townsend area deprivation index was derived from the postcode of residence using aggregated data on unemployment, car and home ownership, and household overcrowding.^30^ Dietary intake was assessed through questions about the frequency of consumption of specific food items the last year, including fruits, vegetables, red and processed meat, and oily fish. Physician‐diagnosed medical conditions were self‐reported at baseline.^25^ These health conditions were used to derive a multimorbidity count, including 43 health conditions.^31^, ^32^, ^33^ Physical activity levels over a typical week were self‐reported using the International Physical Activity Questionnaire and were reported as the metabolic equivalent of task (MET) per week.^34^ Time spent in discretionary sedentary behaviors was derived from the questionnaire and included time spent in front of a television or computer screen or leisure time driving. Further details of these measurements can be obtained in the UK Biobank online protocol.^29^

### Ethics

All participants provided written informed consent before enrolment in the study, which was conducted in accordance with the Declaration of Helsinki. The study was approved by the National Health Service National Research Ethics Service (reference 11/NW/0382).

### Statistical analyses

Descriptive characteristics of the cohort are presented by tertiles of VAI. Continuous variables are presented as means and standard deviations, and categorical variables are presented as frequencies and percentages.

Cox proportional hazard models were used to investigate the associations between the VAI, expressed as sex‐specific tertiles, and incident cancer at 23 sites and all cancers combined. Risk estimates were reported as hazard ratios (HRs) and 95% confidence intervals (CIs). Follow‐up time was used as the time scale for the Cox regression analysis. Individuals who had cancer at baseline were removed (*n* = 41,404); and, to reduce the potential for reverse causality and survival bias, a landmark analysis was performed with follow‐up commencing 2 years after recruitment. To assess the potential non‐linear associations between VAI and the risk of cancer, we applied penalized splines.

Analyses were adjusted hierarchically using three statistical models: model 1 was adjusted for sociodemographic variables only (age, deprivation, ethnicity, income), model 2 was adjusted for model 1 variables plus lifestyle factors (smoking, diet intake of alcohol, fruits and vegetables, red and processed meat, and oily fish, physical activity and sedentary time), and model 3 was adjusted for model 2 variables plus multimorbidity count. Age of menarche and hormone‐replacement and contraceptive use were also added to the models for female cancers (breast, ovarian, cervical, and uterine cancer). For breast cancer, the analyses were repeated stratified by menopausal status (premenopausal and postmenopausal).

We calculated the Harrell C‐index (which estimates the probability of concordance between observed and predicted responses)^35^ to compare the predictive ability between the BMI and the VAI using model 2. We first fitted model 2 with BMI as the adiposity exposure and then replaced the BMI with the VAI to assess whether the C‐index was statistically different depending on the adiposity marker used. All analyses were performed using R statistical software, version 3.6.2 (The R Project for Statistical Computing), with the package *survival*.

## RESULTS

After excluding 75,577 individuals who had missing data on the exposure and covariates, 385,477 participants (53.3% women; mean age, 56.3 years) from the UK Biobank cohort were included in this study (see Figure S1). The characteristics of participants who were included versus those who were excluded are presented in Table S2. The median follow‐up was 8.2 years (interquartile range, 7.3–8.9 year) after the 2‐year landmark period. Overall, 47,882 individuals developed cancer over the follow‐up period.

Table 1 summarizes the main characteristics of the participants by sex‐specific VAI tertiles. In summary, individuals in the highest tertile, compared with those in the lowest tertile, were older (aged 56.9 vs. 55.3 years), were more likely to be from a deprived area (34.8% vs. 31.4%), and were more likely to be smokers (13.2% vs. 8.2%). They also had higher levels of CRP (3.21 ± 4.37 vs. 1.87 ± 3.95 mg/L), engaged in less physical activity, and had a higher prevalence of multimorbidity (69.9% vs. 55.1%). There was minimal difference between the individuals who were included and those who were excluded from the analysis, as presented in Table S2. The correlations between the VAI, the BMI, and each of the markers used to derive the VAI are presented in Figure S2. The correlation (*r*) of the VAI with the BMI was modest (*r* = 0.33), and TGs showed the strongest correlation with the VAI (*r* = 0.93). The correlation between the VAI and the CRP level was weak (*r* = 0.11; see Figure S2).

**TABLE 1:** Cohort characteristic by visceral adiposity index sex‐specific tertiles.

Characteristic	No. (%) or Mean ± SD^a^			
	Lower tertile	Middle tertile	Higher tertile	Overall
Total	129,776 (33.7)	128,514 (33.3)	127,187 (33.0)	385,477 (100.0)
Sex				
Women	78,013 (60.1)	68,748 (53.5)	58,728 (46.2)	205,489 (53.3)
Men	51,763 (39.9)	59,766 (46.5)	68,459 (53.8)	179,988 (46.7)
Age, years	55.3 ± 8.22	56.7 ± 8.07	56.9 ± 7.92	56.3 ± 8.10
Education				
College or university degree	55,741 (43.0)	47,960 (37.3)	42,553 (33.5)	146,254 (37.9)
A levels/AS levels or equivalent	15,572 (12.0)	14,383 (11.2)	13,478 (10.6)	43,433 (11.3)
O levels/GCSEs or equivalent	26,906 (20.7)	27,680 (21.5)	27,458 (21.6)	82,044 (21.3)
SEs or equivalent/NVQ or HND or HNC or equivalent	13,787 (10.6)	15,630 (12.2)	16,910 (13.3)	46,327 (12.0)
Missing	17,770 (13.7)	22,861 (17.8)	26,788 (21.1)	67,419 (17.5)
Townsend deprivation index				
Lower deprivation	45,459 (35.0)	44,180 (34.4)	40,830 (32.1)	130,469 (33.8)
Middle deprivation	43,594 (33.6)	43,465 (33.8)	42,123 (33.1)	129,182 (33.5)
Higher deprivation	40,723 (31.4)	40,869 (31.8)	44,234 (34.8)	125,826 (32.6)
Ethnicity				
White	122,497 (94.4)	121,878 (94.8)	120,622 (94.8)	364,997 (94.7)
Mixed	1960 (1.5)	1918 (1.5)	1836 (1.4)	5714 (1.5)
South Asian	1577 (1.2)	2512 (2.0)	3443 (2.7)	7532 (2.0)
Black	3289 (2.5)	1818 (1.4)	918 (0.7)	6025 (1.6)
Chinese	453 (0.3)	388 (0.3)	368 (0.3)	1209 (0.3)
Height, meters	1.7 ± 0.09	1.7 ± 0.09	1.7 ± 0.10	1.7 ± 0.09
Weight, kg	71.4 ± 13.34	78.4 ± 15.12	85.0 ± 16.18	78.2 ± 15.92
Waist, cm	83.0 ± 11.59	90.6 ± 12.26	97.4 ± 12.35	90.3 ± 13.44
Body fat, %	28.9 ± 8.24	31.7 ± 8.43	33.2 ± 8.35	31.3 ± 8.53
Body mass index, kg/m^2^	25.2 ± 3.87	27.5 ± 4.52	29.6 ± 4.82	27.4 ± 4.76
VAI	0.85 ± 0.23	1.69 ± 0.29	3.89 ± 1.86	2.1 ± 1.68
Smoking				
Never	76,499 (58.9)	71,414 (55.6)	64,660 (50.8)	212,573 (55.1)
Previous	42,590 (32.8)	44,114 (34.3)	45,777 (36.0)	132,481 (34.4)
Current	10,687 (8.2)	12,986 (10.1)	16,750 (13.2)	40,423 (10.5)
Alcohol intake				
Daily or almost daily	32,064 (24.7)	25,743 (20.0)	21,071 (16.6)	78,878 (20.5)
Three or four times a week	34,215 (26.4)	30,102 (23.4)	25,935 (20.4)	90,252 (23.4)
Once or twice a week	32,321 (24.9)	33,858 (26.3)	33,885 (26.6)	100,064 (26.0)
Once or three times a month	12,314 (9.5)	14,386 (11.2)	16,324 (12.8)	43,024 (11.2)
Special occasions only	11,142 (8.6)	14,668 (11.4)	17,561 (13.8)	43,371 (11.3)
Never	7720 (5.9)	9757 (7.6)	12,411 (9.8)	29,888 (7.8)
Dietary intake				
Fruit and vegetable intake, portions/day	2.0 ± 0.82	2.0 ± 0.83	1.9 ± 0.83	2.0 ± 0.83
Red meat, portions/week	2.0 ± 1.41	2.1 ± 1.44	2.2 ± 1.48	2.1 ± 1.45
Processed meat, portions/week	1.8 ± 1.06	1.9 ± 1.05	2.0 ± 1.06	1.9 ± 1.06
Oily fish, portions/week	1.7 ± 0.92	1.6 ± 0.92	1.5 ± 0.93	1.6 ± 0.93
Physical activity				
Sedentary time, hours/day	4.7 ± 2.11	5.1 ± 2.24	5.4 ± 2.42	5.0 ± 2.28
Walking for pleasure	96,315 (74.2)	91,702 (71.4)	85,172 (67.0)	273,189 (70.9)
Other exercises	16,927 (13.0)	15,855 (12.3)	15,150 (11.9)	47,932 (12.4)
Strenuous sports	1233 (1.0)	980 (0.8)	924 (0.7)	3137 (0.8)
Light DIY	6763 (5.2)	8547 (6.7)	10,558 (8.3)	25,868 (6.7)
Heavy DIY	2614 (2.0)	3380 (2.6)	3870 (3.0)	9864 (2.6)
Missing	5924 (4.6)	8050 (6.3)	11,513 (9.1)	25,487 (6.6)
Multimorbidity				
No	58,316 (44.9)	47,432 (36.9)	38,310 (30.1)	144,058 (37.4)
Yes	71,460 (55.1)	81,082 (63.1)	88,877 (69.9)	241,419 (62.6)
C‐reactive protein, mg/L	1.87 ± 3.95	2.59 ± 4.33	3.21 ± 4.37	2.55 ± 4.26

Abbreviations: A/AS levels, General Certificate of Education Advanced/Advanced Subsidiary levels; DIY, do‐it‐yourself; GCSEs, General Certificates Secondary Education; HNC, Higher National Certificate; HND, Higher National Diploma; NVQ, National Vocational Qualification; O levels, General Certificate of Education Ordinary levels; SD, standard deviation; VAI, visceral adiposity index.

^a^ Data are presented as the number or % of participants for continuous and categorical variables, respectively. Continuous variables are presented as the mean ± SD by sex‐specific tertiles of the VAI (men: low, <1.35; middle, 1.35–2.47; high, ≥2.48; women: low, <1.15; middle, 1.15–2.07; high, ≥2.08).

**FIGURE 1:**
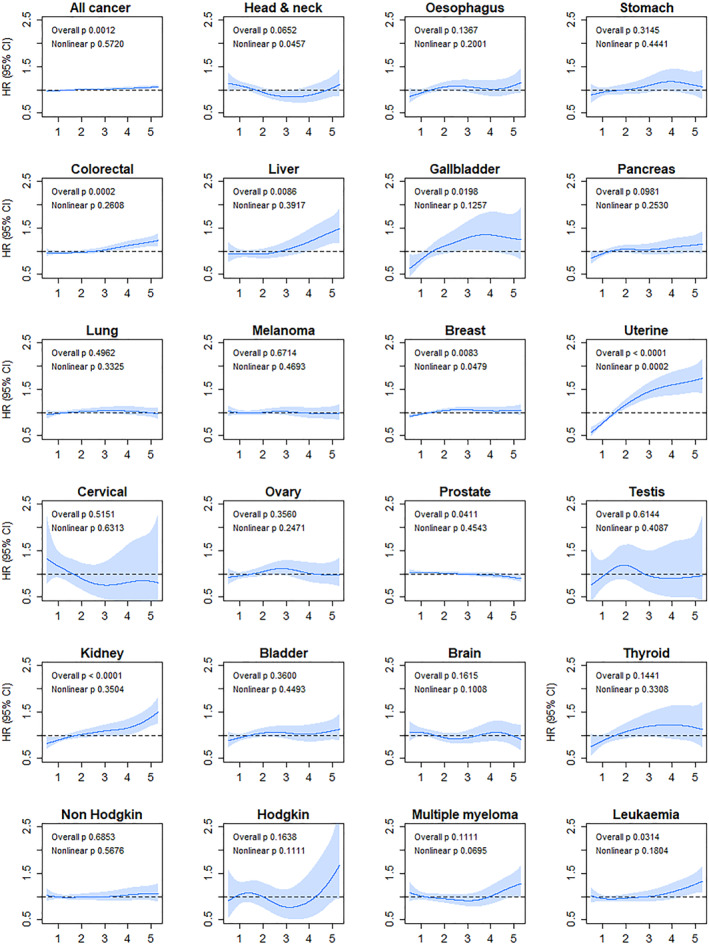
Association between the VAI and cancer sites in a fully adjusted model. VAI was sex‐standardised analyses were adjusted for age, income, deprivation, ethnicity, diet (red and processed meat, fruits and vegetables, oily fish, and alcohol), smoking, sedentary behavior and physical activity, and comorbidity. CI indicates confidence interval; HR, hazard ratio; VAI, visceral adiposity index.

Associations between sex‐specific VAI tertiles and incident cancer are detailed in Table 2. In the fully adjusted model, the VAI was associated with six cancer sites. Individuals in the highest tertile, compared with those in the lowest tertile, had a higher risk of uterine (HR, 2.09; 95% CI, 1.76–2.49), gallbladder (HR, 1.83; 95% CI, 1.26–2.66), kidney (HR, 1.39; 95% CI,1.18–1.64), liver (HR, 1.25; 95% CI, 1.00–1.56), colorectal (HR, 1.14; 95% CI, 1.05–1.24 [especially proximal colonic]), and breast (HR, 1.11; 95% CI, 1.03–1.19) cancers and higher risk of all‐cause cancer (HR, 1.05; 95% CI, 1.03–1.09; Table 2). Similar results were observed for the minimally adjusted models (see Table S3). However, when the BMI was included in the model, only esophageal, gallbladder, and proximal colon cancers remained significant (see Table S4). As shown in Figure 1, there was no evidence of nonlinear associations for the VAI fitted as a continuous variable and the risk of cancer at any of the 23 sites.

**TABLE 2:** Association between the visceral adiposity index (VAI) and specific cancer sites across sex‐specific tertiles of the VAI in a fully adjusted model.^a^

		Lowest tertile		Middle tertile			Highest tertile			Trend		
Cancer	Total no.	No. of events	HR (95% CI)	No. of events	HR (95% CI)	*p*	No. of events	HR (95% CI)	*p*	No. of events	HR (95% CI)	*p*
All cause	379,848	9362	1.00 (Ref.)	10,751	1.04 (1.01–1.07)	.006	11,443	1.05 (1.03–1.09)	< .001	31,556	1.03 (1.01–1.04)	< .001
Brain	385,388	204	1.00 (Ref.)	192	0.84 (0.69–1.03)	.094	205	0.86 (0.70–1.05)	.132	601	0.93 (0.84–1.03)	.139
Head and neck	385,371	207	1.00 (Ref.)	232	1.02 (0.85–1.24)	.811	216	0.85 (0.70–1.04)	.118	655	0.92 (0.84–1.02)	.109
Esophagus	385,375	184	1.00 (Ref.)	273	1.16 (0.96–1.40)	.121	336	1.19 (0.99–1.43)	.065	793	1.08 (0.99–1.19)	.080
Lung	385,114	679	1.00 (Ref.)	933	1.08 (0.98–1.20)	.114	1178	1.09 (0.99–1.21)	.070	2790	1.04 (0.99–1.09)	.087
Liver	385,425	126	1.00 (Ref.)	160	0.97 (0.77–1.23)	.802	243	1.25 (1.00–1.56)	.049	529	1.14 (1.02–1.27)	.026
Stomach	385,407	131	1.00 (Ref.)	173	1.09 (0.87–1.37)	.468	205	1.15 (0.92–1.45)	.213	509	1.07 (0.96–1.20)	.215
Pancreas	385,375	261	1.00 (Ref.)	355	1.17 (1.00–1.38)	.052	388	1.15 (0.98–1.36)	.087	1004	1.07 (0.99–1.16)	.109
Gallbladder	385,452	42	1.00 (Ref.)	80	1.63 (1.12–2.37)	.011	100	1.83 (1.26–2.66)	.001	222	1.32 (1.11–1.57)	.002
Bladder	385,342	191	1.00 (Ref.)	236	1.01 (0.84–1.23)	.891	289	1.11 (0.92–1.34)	.289	716	1.06 (0.96–1.16)	.264
Kidney	385,339	233	1.00 (Ref.)	346	1.24 (1.05–1.47)	.011	432	1.39 (1.18–1.64)	< .001	1011	1.17 (1.08–1.27)	< .001
Colorectal	384,798	1063	1.00 (Ref.)	1226	1.05 (0.97–1.14)	.260	1391	1.14 (1.05–1.24)	.001	3680	1.07 (1.03–1.12)	.001
Colon	385,025	711	1.00 (Ref.)	824	1.05 (0.95–1.16)	.354	966	1.19 (1.07–1.31)	.001	2501	1.09 (1.04–1.15)	.001
Proximal	385,281	358	1.00 (Ref.)	440	1.09 (0.95–1.25)	.239	527	1.25 (1.09–1.44)	.001	1325	1.12 (1.05–1.20)	.001
Distal	385,254	312	1.00 (Ref.)	350	1.03 (0.88–1.20)	.732	395	1.12 (0.96–1.30)	.161	1057	1.06 (0.98–1.14)	.153
Rectum	385,235	414	1.00 (Ref.)	495	1.09 (0.95–1.24)	.204	510	1.07 (0.94–1.22)	.324	1419	1.03 (0.97–1.10)	.347
Thyroid	385,421	82	1.00 (Ref.)	85	1.06 (0.78–1.44)	.726	105	1.31 (0.97–1.77)	.083	272	1.15 (0.98–1.34)	.079
Lymphatic	385,030	903	1.00 (Ref.)	1006	0.99 (0.91–1.09)	.890	1074	1.01 (0.93–1.11)	.754	2983	1.01 (0.96–1.06)	.741
Leukemia	385,349	259	1.00 (Ref.)	304	1.03 (0.87–1.22)	.719	350	1.13 (0.95–1.33)	.158	913	1.06 (0.98–1.16)	.148
Multiple myeloma	385,403	198	1.00 (Ref.)	218	0.98 (0.81–1.20)	.868	223	0.98 (0.80–1.19)	.834	639	0.99 (0.90–1.09)	.837
Melanoma	385,153	612	1.00 (Ref.)	603	0.95 (0.85–1.06)	.369	627	1.01 (0.90–1.13)	.895	1842	1.00 (0.95–1.06)	.888
Non‐Hodgkin lymphoma	385,249	435	1.00 (Ref.)	472	0.98 (0.86–1.12)	.780	498	1.00 (0.87–1.14)	.966	1405	1.00 (0.93–1.07)	.976
Hodgkin lymphoma	385,458	28	1.00 (Ref.)	30	0.85 (0.51–1.43)	.543	33	0.76 (0.45–1.28)	.295	91	0.87 (0.67–1.13)	.299
Prostate	178,801	2095	1.00 (Ref.)	2466	1.01 (0.95–1.07)	.856	2500	0.95 (0.89–1.01)	.085	7061	0.97 (0.94–1.00)	.072
Testis	179,975	14	1.00 (Ref.)	23	1.46 (0.75–2.85)	.271	22	1.18 (0.59–2.34)	.637	59	1.06 (0.77–1.46)	.721
Breast	204,270	1955	1.00 (Ref.)	1923	1.09 (1.02–1.16)	.008	1689	1.11 (1.03–1.19)	.003	5567	1.05 (1.02–1.09)	.003
Breast, premenopausal	51,012	587	1.00 (Ref.)	380	1.03 (0.91–1.18)	0.607	236	1.02 (0.87–1.19)	.846	1203	1.01 (0.94–1.09)	.759
Breast, postmenopausal	121,990	1128	1.00 (Ref.)	1257	1.12 (1.03–1.21)	.007	1120	1.12 (1.03–1.22)	.009	3505	1.06 (1.01–1.11)	.009
Uterine	205,303	214	1.00 (Ref.)	322	1.55 (1.30–1.85)	< .001	403	2.09 (1.76–2.49)	< .001	939	1.43 (1.32–1.56)	< .001
Ovary	205,353	222	1.00 (Ref.)	216	1.00 (0.82–1.21)	.976	233	1.15 (0.95–1.39)	.160	671	1.07 (0.97–1.18)	.161
Cervix	205,461	29	1.00 (Ref.)	25	0.94 (0.54–1.62)	.816	15	0.62 (0.32–1.19)	.147	69	0.80 (0.58–1.10)	.164

Abbreviations: CI, confidence interval; HR, hazard ratio; Ref., reference category.

^a^ Data are presented as HRs and 95% CIs. The reference group was individuals in the lowest tertile. Analyses were adjusted for age, income, deprivation, ethnicity, diet (red and processed meat, fruits and vegetables, oily fish, and alcohol), smoking, sedentary behavior and physical activity, and the multimorbidity count.

The calculated Harrell C‐indices demonstrated little difference in the prediction of all‐cause and site‐specific cancers when using the VAI instead of the BMI, except for breast cancer and all‐cause cancer, for which prediction was better using the BMI than the VAI (Table 3).

**TABLE 3:** Comparative C‐indices of body mass index and visceral adiposity index in predicting cancer incidence risk.

	BMI (95% CI)	VAI (95% CI)	C‐index delta (95% CI)	*p*
All cause	0.6500 (0.6469–0.6530)	0.6493 (0.6463–0.6524)	−0.0006 (−0.0009, −0.0004)	< .001
Brain	0.6289 (0.6063–0.6510)	0.6291 (0.6064–0.6514)	0.0002 (−0.0020, 0.0024)	.856
Head and neck	0.6785 (0.6559–0.7003)	0.6794 (0.6570–0.7003)	0.0009 (−0.0025, 0.0042)	.616
Esophagus	0.7759 (0.7590–0.7919)	0.7728 (0.7559–0.7891)	−0.0031 (−0.0066, 0.0004)	.083
Liver	0.7541 (0.7330–0.7740)	0.7533 (0.7319–0.7747)	−0.0007 (−0.0058, 0.0044)	.786
Stomach	0.7415 (0.7196–0.7621)	0.7357 (0.7136–0.7576)	−0.0057 (−0.0118, 0.0003)	.063
Pancreas	0.7029 (0.6872–0.7182)	0.7005 (0.6847–0.7162)	−0.0024 (−0.0054, 0.0006)	.118
Lung	0.8301 (0.8219–0.8379)	0.8297 (0.8216–0.8376)	−0.0004 (−0.0011, 0.0003)	.261
Melanoma	0.6235 (0.6108–0.6360)	0.6236 (0.6109–0.6361)	0.0001 (−0.0013, 0.0016)	.848
Non‐Hodgkin lymphoma	0.6534 (0.6393–0.6673)	0.6535 (0.6393–0.6674)	0.0001 (−0.0004, 0.0005)	.779
Multiple myeloma	0.6764 (0.6549–0.6971)	0.6760 (0.6546–0.6967)	−0.0003 (−0.0029, 0.0023)	.804
Lymphatic	0.6629 (0.6532–0.6724)	0.6628 (0.6531–0.6725)	−0.0001 (−0.0010, 0.0009)	.905
Leukemia	0.6865 (0.6693–0.7031)	0.6863 (0.6690–0.7034)	−0.0002 (−0.0029, 0.0026)	.896
Thyroid	0.6390 (0.6049–0.6717)	0.6387 (0.6056–0.6687)	−0.0003 (−0.0093, 0.0088)	.951
Gallbladder	0.7150 (0.6808–0.7469)	0.7082 (0.6734–0.7426)	−0.0068 (−0.0154, 0.0018)	.124
Bladder	0.7966 (0.7805–0.8117)	0.7953 (0.7792–0.8105)	−0.0013 (−0.0032, 0.0006)	.180
Kidney	0.7136 (0.6987–0.7281)	0.7088 (0.6939–0.7235)	−0.0048 (−0.0098, 0.0002)	.059
Prostate	0.6875 (0.6822–0.6928)	0.6875 (0.6822–0.6928)	0.0000 (−0.0005, 0.0004)	.842
Testis	0.6895 (0.6175–0.7533)	0.6883 (0.6155–0.7550)	−0.0011 (−0.0048, 0.0026)	.551
Colorectal	0.6720 (0.6634–0.6806)	0.6718 (0.6631–0.6803)	−0.0003 (−0.0017, 0.0011)	.696
Colon	0.6706 (0.6601–0.6809)	0.6704 (0.6600–0.6807)	−0.0002 (−0.0022, 0.0019)	.878
Distal	0.6543 (0.6375–0.6706)	0.6508 (0.6341–0.6670)	−0.0035 (−0.0072, 0.0003)	.069
Proximal	0.6933 (0.6796–0.7067)	0.6943 (0.6806–0.7077)	0.0010 (−0.0017, 0.0037)	.462
Rectum	0.6797 (0.6655–0.6936)	0.6790 (0.6649–0.6927)	−0.0007 (−0.0021, 0.0008)	.359
Hodgkin	0.6889 (0.6329–0.7400)	0.6895 (0.6337–0.7395)	0.0005 (−0.0057, 0.0068)	.866
Breast	0.5519 (0.5442–0.5595)	0.5473 (0.5396–0.5548)	−0.0046 (−0.0078, −0.0015)	.004
Breast, postmenopausal	0.5519 (0.5442–0.5595)	0.5473 (0.5396–0.5548)	−0.0046 (−0.0078, −0.0015)	.004
Breast, premenopausal	0.5519 (0.5442–0.5595)	0.5473 (0.5396–0.5548)	−0.0046 (−0.0078, −0.0015)	.004
Ovary	0.6164 (0.5951–0.6372)	0.6161 (0.5947–0.6370)	−0.0003 (−0.0033, 0.0026)	.830
Uterine	0.7195 (0.7018–0.7366)	0.6737 (0.6568–0.6885)	−0.0458 (−0.0590, −0.0327)	< .0001
Cervix	0.6792 (0.6157–0.7366)	0.6983 (0.6385–0.7422)	0.0192 (−0.0050, 0.0434)	.120

Abbreviations: BMI, body mass index; CI, confidence interval; VAI, visceral adiposity index.

## DISCUSSION

In this prospective cohort study of greater than 380,000 participants, we investigated the association between the VAI and incident cancers. Our findings revealed that a higher VAI was associated with a higher risk of six cancer sites (uterine, gallbladder, kidney, liver, colorectal, and breast) and of all‐cause cancer. To our knowledge, this is the first cohort study reporting longitudinal associations between the VAI and different incident cancers.

Our findings corroborate previous research demonstrating associations between the VAI and specific cancer types.^21^, ^22^, ^23^ For instance, a previous case–control study conducted by Godinho‐Mota et al.^21^ that included 342 women (116 with breast cancer and 226 as controls) reported that women who had a higher VAI (≥1.72) had 91% higher odds of breast cancer (odds ratio, 1.91; 95% CI, 1.17–3.13) than women with a VAI <1.72.^21^ In relation to CRC, Okamura et al.^22^ conducted a study of 27,921 participants (16,343 men and 11,487 women), of whom 116 developed CRC over a median of 4.4 years. Compared with individuals in the lowest tertile for VAI (<0.44), those in the highest tertile (>1.13) had a 78% higher risk of CRC (HR, 1.78; 95% CI, 1.05–3.02) after adjustment for confounders, including BMI. Our study also demonstrated significant associations with breast cancer and CRC, but the magnitude of risk was lower than that in the previous two studies. This could be explained using different cutoff points for tertiles of the VAI because we used a sex‐specific cutoff point for the VAI to account for sex differences on the VAI. Differences on adjustment could also be explained by the covariates included in the models. No other studies were identified that investigated the association between the VAI and cancer. Therefore, our study extends and provides novels findings on the association between the VAI and uterine, gallbladder, kidney, and liver cancers.

The VAI has demonstrated some good‐to‐strong correlation with visceral fat from dual x‐ray absorptiometry and insulin sensitivity, which was closely linked to cardiometabolic risk in previous studies.^10^ The connection between visceral fat and cancer involves a range of complex mechanisms.^36^ These include a decrease in adiponectin levels and an increase in adipocytokines^37^, ^38^ like tumor necrosis factor‐α and interleukin,^39^ contributing to insulin resistance^40^, ^41^ and oxidative stress.^42^ Moreover, the association of visceral fat with metabolic syndrome escalates the likelihood of common cancers.^8^, ^43^ A key mechanism also involves insulin resistance,^9^ highlighted by elevated serum C‐peptide levels, a sign of increased insulin production found in insulin‐resistance cases, which has been specifically associated with CRC development.^44^ Insulin resistance is notably implicated in CRC pathogenesis, in which it triggers hyperinsulinemia and boosts insulin‐like growth factors. These factors then stimulate the I3K/Akt/mTOR/S6K signaling pathway, which is crucial in cancer progression.^45^ Furthermore, the VAI's relation with higher levels of CRP, a widely used inflammation marker in clinical screenings, supports the link between adiposity and cancer risk, as demonstrated in our study. This association is reinforced by both observational studies and Mendelian randomization studies, which demonstrate that elevated levels of CRP correlate with an increased risk of cancer.^46^

However, our study is not exempt from limitations. The UK Biobank study is not a representative sample of the older adult population in the United Kingdom, so summary statistics should not be generalized to the general population. However, relative risks derived from UK Biobank are consistent with more representative population cohorts.^47^ The anthropometric measurements used in the study were obtained by trained staff using standardized protocols, thus minimizing the chance of measurement error and misclassification. However, there are several limitations that should be taken into account. Reverse causation is a concern in any epidemiological study of the association between adiposity and cancer. However, to minimize the effect of reverse causation in our study, we excluded baseline cancers and all cancers that were diagnosed within the first 2‐years of follow‐up. Residual confounding is also possible, although we have adopted a comprehensive adjustment scheme. We used data from hospital admissions and death certificates to ascertain incident cases. Whilst routine data may be incomplete or inaccurate, there is no reason to believe that systematic error will occur. Although UK Biobank is a large observational study, some cancers are rare, which limits our power to identify some associations with the VAI.

In conclusion, we have demonstrated that the VAI is associated with different cancer sites and all‐cause cancer. However, our findings provide evidence that the VAI does not improve cancer risk prediction beyond the BMI. Considering that the BMI is cheaper, widely used, and easy to implement, this marker should continue to be used to identify individuals at higher risk of developing cancer among those who are seemingly healthy to support prevention and early detection.^36^

## AUTHOR CONTRIBUTIONS

**Solange Parra‐Soto**: Conceptualization, writing–original draft, writing–review and editing, formal analysis, and methodology. **Jirapitcha Boonpor**: Writing–review and editing. **Nathan Lynskey**: Writing–review and editing. **Carolina Araya**: Writing–review and editing. **Frederick Ho**: Writing–review and editing, project administration, and supervision. **Jill P. Pell**: Conceptualization, writing–review and editing, supervision, and project administration. **Carlos Celis‐Morales**: Conceptualization, writing–review and editing, formal analysis, supervision, and project administration.

## CONFLICT OF INTEREST STATEMENT

The authors declared no conflicts of interest.

## Supporting information

Supplementary Material

## Data Availability

The data that support the findings of this study are available on request from the corresponding author. The data are not publicly available because of privacy or ethical restrictions.
