# Supplementary material for: Association between visceral adiposity index and cancer risk in the UK Biobank cohort
Source: Cancer. 2024 Oct 3;131(1):e35576. doi: 10.1002/cncr.35576 (PMC11694164; doi:10.1002/cncr.35576)
Supplement: Supplementary file 1 — Supplementary Material [file CNCR-131-0-s001.docx]

**Supplementary Material: Parra-Soto et al., Visceral adiposity index and its association with cancer risk in the UK Biobank cohort.**

People with missing in exposure and covariates:

(N= 75,577)

VAI= 67,472

Covariates = 8,105

Exclusion of Participants with Pre-Existing Cancer at Baseline=41,404

Participants finally included in the study

**n=385,477**

Participants included

461,054

Participants recruited in the UK Biobank study

502,458

**Figure S1**: Flowchart participants included.


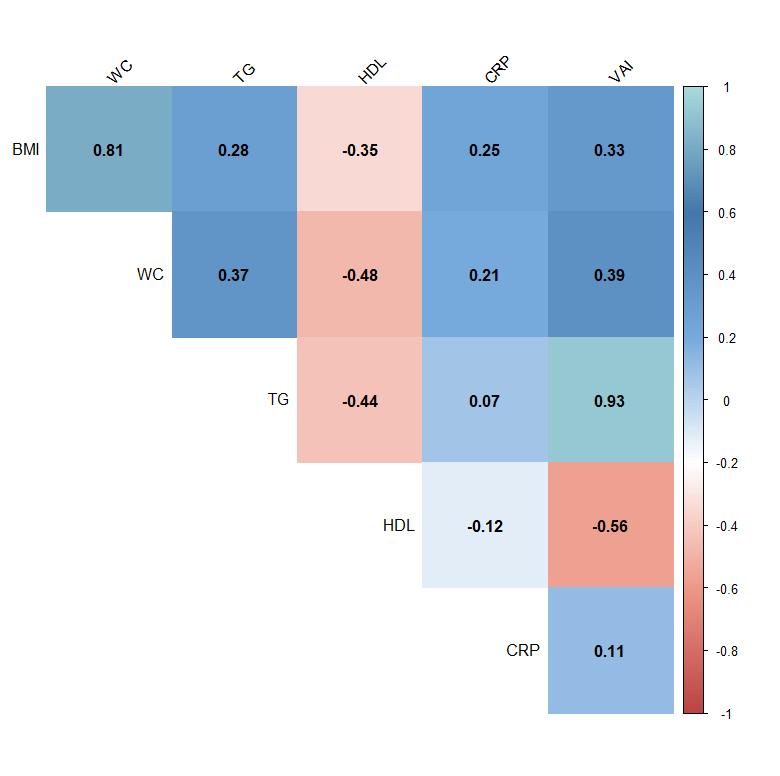


**Figure S2**: Correlation Among BMI, Waist Circumference (WC), Triglycerides (TG), HDL Cholesterol, and Visceral Adiposity Index (VAI)

BMI: body mass index, WC: waist circumference, TG: triglycerides, HDL: high-density lipoprotein cholesterol, CRP: C-reactive protein, VAI: visceral adiposity index.

Table S1: Sex-Specific Tertiles of VAI and Their Corresponding Cutoff Points.

| Sex specific tertiles | Lower | Middle | Higher |
| --- | --- | --- | --- |
| Men | <1.35  N=51,757 | 1.35-2.47  N=59,760 | ≥2.48  N=68,454 |
| Women | <1.15  N=78,005 | 1.15-2.07  N=68,742 | ≥2.08  N=58,723 |

Table S2: Cohort Characteristics of Participants Included and Excluded from the Study due to missing data.

|  | Excluded | Overall |
| --- | --- | --- |
| N (%) | 116,970 | 385,477 |
| Sex |  |  |
| Females | 67847 (58.0%) | 205,489 (53.3%) |
| Males | 49108 (42.0%) | 179,988 (46.7%) |
| Age Mean (SD) | 57.36 (8.01) | 56.3 (8.10) |
| Education |  |  |
| College or University degree | 40685 (45.7%) | 146,254 (37.9%) |
| A levels/AS levels or equivalent | 11881 (13.4%) | 43,433 (11.3%) |
| O levels/GCSEs or equivalent | 23135 (26.0%) | 82,044 (21.3%) |
| SEs or equivalent/NVQ or HND or HNC or equivalent | 13281 (14.9%) | 46,327 (12.0%) |
| Missing |  | 67419 (17.5%) |
| Townsend deprivation index |  |  |
| Lower deprivation | 36933 (31.7%) | 130,469 (33.8%) |
| Middle deprivation | 37992 (32.7%) | 129,182 (33.5%) |
| Higher deprivation | 41407 (35.6%) | 125,826 (32.6%) |
| Ethnicity |  |  |
| White | 107633 (94.3%) | 364,997 (94.7%) |
| Mixed | 1800 (1.6%) | 5,714 (1.5%) |
| South Asian | 2348 (2.1%) | 7,532 (2.0%) |
| Black | 2034 (1.8%) | 6,025 (1.6%) |
| Chinese | 364 (0.3%) | 1,209 (0.3%) |
| Height (m) | 1.68 (0.09) | 1.7 (0.09) |
| Weight (Kg) | 77.58 (16.01) | 78.2 (15.92) |
| Waist circumference (cm) | 90.33 (13.64) | 90.3 (13.44) |
| Body fat % (%) | 32.08 (8.58) | 31.3 (8.53) |
| Body Mass index (kg/m^2^) | 27.50 (4.93) | 27.4 (4.76) |
| VAI | 2.27 (1.78) | 2.1 (1.68) |
| Smoking |  |  |
| Never | 60918 (53.4%) | 212,573 (55.1%) |
| Previous | 40559 (35.6%) | 132,481 (34.4%) |
| Current | 12545 (11.0%) | 40,423 (10.5%) |
| Alcohol intake |  |  |
| Daily or almost daily | 22884 (19.8%) | 78,878 (20.5%) |
| 3-4 times a week | 25170 (21.8%) | 90,252 (23.4%) |
| Once or twice a week | 29211 (25.3%) | 100,064 (26.0%) |
| 1-3 times a month | 12819 (11.1%) | 43,024 (11.2%) |
| Special occasions only | 14627 (12.7%) | 43,371 (11.3%) |
| Never | 10743 (9.3%) | 29,888 (7.8%) |
| Fruit and vegetable intake (portion/day) | 2.0 (0.73) | 2.0 (0.83) |
| Red meat (portion/week) | 2.1 (1.45) | 2.1 (1.45) |
| Processed meat (portion/week) | 1.8 (1.07) | 1.9 (1.06) |
| Oily fish (portion/week) | 1.7 (0.94) | 1.6 (0.93) |
| Sedentary time (hr/day) | 4.9 (2.36) | 5.0 (2.28) |
| Physical activity |  |  |
| Walking for pleasure | 77710 (75.8%) | 273,189 (70.9%) |
| Other exercises | 13225 (12.9%) | 47,932 (12.4%) |
| Strenuous sports | 734 (0.7%) | 3,137 (0.8%) |
| Light DIY | 7944 (7.8%) | 25,868 (6.7%) |
| Heavy DIY | 2841 (2.8%) | 9,864 (2.6%) |
| Missing | 77710 (75.8%) | 25487 (6.6%) |
| Multimorbidity |  |  |
| No | 29406 (25.1%) | 144,058 (37.4%) |
| Yes | 87564 (74.9%) | 241,419 (62.6%) |

Data are presented as number of participants and their percentage (%) for categorical variables. Continuous variables are presented as mean and standard deviation.

**Table S3:** Association Between VAI and Cancer Sites: Comparing Model 1 and Model 2.

|  |  | Lowest tertile | | Middle tertile | | | Highest tertile | | | Trend | | |
| --- | --- | --- | --- | --- | --- | --- | --- | --- | --- | --- | --- | --- |
| Model 1 |  |  | |  | | |  | | |  |  |  |
| cancer | **Total** | **Event** | **HR 95% CI** | **Event** | **HR 95% CI** | **P value** | **Event** | **HR 95% CI** | **P value** | **Event** | **HR 95% CI** | **P value** |
| All cause | 379,848 | 9,362 | 1.00 (Ref.) | 10,751 | **1.06 (1.03; 1.09)** | **<0.001** | 11,443 | **1.10 (1.07; 1.13)** | **<0.001** | 31,556 | **1.05 (1.03; 1.06)** | **<0.001** |
| Brain | 385,388 | 204 | 1.00 (Ref.) | 192 | 0.85 (0.70; 1.04) | 0.109 | 205 | 0.87 (0.71; 1.06) | 0.158 | 601 | 0.93 (0.84; 1.03) | 0.166 |
| Head & neck | 385,371 | 207 | 1.00 (Ref.) | 232 | 1.03 (0.85; 1.24) | 0.757 | 216 | 0.89 (0.73; 1.08) | 0.231 | 655 | 0.94 (0.86; 1.04) | 0.219 |
| Oesophagus | 385,375 | 184 | 1.00 (Ref.) | 273 | **1.26 (1.04; 1.52)** | **0.016** | 336 | **1.44 (1.20; 1.72)** | **<0.001** | 793 | **1.19 (1.09; 1.30)** | **<0.001** |
| Lung | 385,114 | 679 | 1.00 (Ref.) | 933 | **1.20 (1.09; 1.33)** | **<0.001** | 1,178 | **1.42 (1.29; 1.56)** | **<0.001** | 2,790 | **1.19 (1.14; 1.25)** | **<0.001** |
| Liver | 385,425 | 126 | 1.00 (Ref.) | 160 | 1.08 (0.85; 1.36) | 0.527 | 243 | **1.59 (1.28; 1.97)** | **<0.001** | 529 | **1.28 (1.15; 1.43)** | **<0.001** |
| Stomach | 385,407 | 131 | 1.00 (Ref.) | 173 | 1.15 (0.91; 1.44) | 0.233 | 205 | **1.29 (1.03; 1.60)** | **0.025** | 509 | **1.13 (1.02; 1.26)** | **0.024** |
| Pancreas | 385,375 | 261 | 1.00 (Ref.) | 355 | **1.22 (1.04; 1.43)** | **0.016** | 388 | **1.26 (1.07; 1.47)** | **0.004** | 1,004 | **1.12 (1.03; 1.21)** | **0.006** |
| Gallbladder | 385,452 | 42 | 1.00 (Ref.) | 80 | **1.72 (1.18; 2.50)** | **0.005** | 100 | **2.04 (1.42; 2.94)** | **<0.001** | 222 | **1.39 (1.17; 1.65)** | **<0.001** |
| Bladder | 385,342 | 191 | 1.00 (Ref.) | 236 | 1.05 (0.87; 1.27) | 0.615 | 289 | 1.20 (0.99; 1.44) | 0.057 | 716 | **1.10 (1.00; 1.20)** | **0.050** |
| Kidney | 385,339 | 233 | 1.00 (Ref.) | 346 | **1.32 (1.12; 1.56)** | **0.001** | 432 | **1.58 (1.34; 1.85)** | **<0.001** | 1,011 | **1.25 (1.16; 1.35)** | **<0.001** |
| Colorectal | 384,798 | 1,063 | 1.00 (Ref.) | 1,226 | 1.06 (0.97; 1.15) | 0.194 | 1,391 | **1.16 (1.07; 1.26)** | **<0.001** | 3,680 | **1.08 (1.04; 1.12)** | **<0.001** |
| Colon | 385,025 | 711 | 1.00 (Ref.) | 824 | 1.06 (0.96; 1.18) | 0.227 | 966 | **1.22 (1.11; 1.34)** | **<0.001** | 2,501 | **1.11 (1.05; 1.16)** | **<0.001** |
| Proximal | 385,281 | 358 | 1.00 (Ref.) | 440 | 1.11 (0.97; 1.28) | 0.130 | 527 | **1.32 (1.15; 1.51)** | **<0.001** | 1,325 | **1.15 (1.07; 1.23)** | **<0.001** |
| Distal | 385,254 | 312 | 1.00 (Ref.) | 350 | 1.03 (0.89; 1.20) | 0.691 | 395 | 1.13 (0.97; 1.31) | 0.122 | 1,057 | 1.06 (0.99; 1.14) | 0.116 |
| Rectum | 385,235 | 414 | 1.00 (Ref.) | 495 | 1.08 (0.95; 1.23) | 0.231 | 510 | 1.06 (0.93; 1.21) | 0.391 | 1,419 | 1.03 (0.96; 1.10) | 0.417 |
| Thyroid | 385,421 | 82 | 1.00 (Ref.) | 85 | 1.10 (0.81; 1.49) | 0.535 | 105 | **1.43 (1.07; 1.92)** | **0.017** | 272 | **1.20 (1.03; 1.39)** | **0.016** |
| Lymphatic | 385,030 | 903 | 1.00 (Ref.) | 1,006 | 1.01 (0.93; 1.11) | 0.792 | 1,074 | 1.05 (0.96; 1.15) | 0.256 | 2,983 | 1.03 (0.98; 1.07) | 0.249 |
| Leukaemia | 385,349 | 259 | 1.00 (Ref.) | 304 | 1.05 (0.89; 1.24) | 0.593 | 350 | 1.16 (0.99; 1.37) | 0.069 | 913 | 1.08 (1.00; 1.17) | 0.063 |
| Multiple Myeloma | 385,403 | 198 | 1.00 (Ref.) | 218 | 0.98 (0.81; 1.19) | 0.873 | 223 | 0.98 (0.81; 1.19) | 0.833 | 639 | 0.99 (0.90; 1.09) | 0.835 |
| Melanoma | 385,153 | 612 | 1.00 (Ref.) | 603 | 0.93 (0.83; 1.04) | 0.231 | 627 | 0.97 (0.87; 1.08) | 0.581 | 1,842 | 0.98 (0.93; 1.04) | 0.591 |
| Non-Hodgkin | 385,249 | 435 | 1.00 (Ref.) | 472 | 1.00 (0.88; 1.14) | 0.969 | 498 | 1.03 (0.90; 1.17) | 0.659 | 1,405 | 1.02 (0.95; 1.08) | 0.651 |
| Hodgkin | 385,458 | 28 | 1.00 (Ref.) | 30 | 1.01 (0.60; 1.69) | 0.966 | 33 | 1.08 (0.65; 1.79) | 0.773 | 91 | 1.04 (0.81; 1.34) | 0.770 |
| Prostate | 178,801 | 2,095 | 1.00 (Ref.) | 2,466 | 0.99 (0.93; 1.05) | 0.656 | 2,500 | **0.91 (0.86; 0.96)** | **0.001** | 7,061 | **0.95 (0.93; 0.98)** | **0.001** |
| Testis | 179,975 | 14 | 1.00 (Ref.) | 23 | 1.43 (0.74; 2.78) | 0.291 | 22 | 1.14 (0.58; 2.24) | 0.699 | 59 | 1.04 (0.76; 1.43) | 0.787 |
| Breast | 204,270 | 1,955 | 1.00 (Ref.) | 1,923 | **1.08 (1.01; 1.15)** | **0.022** | 1,689 | **1.08 (1.01; 1.16)** | **0.018** | 5,567 | **1.04 (1.01; 1.08)** | **0.015** |
| Breast Postmenopausal | 121,990 | 1,128 | 1.00 (Ref.) | 1,257 | **1.11 (1.02; 1.20)** | **0.014** | 1,120 | **1.10 (1.01; 1.20)** | **0.026** | 3,505 | **1.05 (1.01; 1.09)** | **0.024** |
| Breast Premenopausal | 51,012 | 587 | 1.00 (Ref.) | 380 | 1.01 (0.89; 1.15) | 0.831 | 236 | 0.97 (0.84; 1.13) | 0.737 | 1,203 | 0.99 (0.92; 1.07) | 0.811 |
| Endometrium | 205,303 | 214 | 1.00 (Ref.) | 322 | **1.58 (1.32; 1.88)** | **<0.001** | 403 | **2.15 (1.82; 2.55)** | **<0.001** | 939 | **1.45 (1.34; 1.58)** | **<0.001** |
| Ovary | 205,353 | 222 | 1.00 (Ref.) | 216 | 1.01 (0.84; 1.22) | 0.914 | 233 | 1.18 (0.98; 1.42) | 0.082 | 671 | 1.09 (0.99; 1.19) | 0.083 |
| Cervix | 205,461 | 29 | 1.00 (Ref.) | 25 | 0.95 (0.55; 1.63) | 0.854 | 15 | 0.64 (0.34; 1.20) | 0.165 | 69 | 0.81 (0.60; 1.10) | 0.183 |
| Model 2 |  |  |  |  |  |  |  |  |  |  |  |  |
|  | **Total** | **Event** | **HR 95% CI** | **Event** | **HR 95% CI** | **P value** | **Event** | **HR 95% CI** | **P value** | **Event** | **HR 95% CI** | **P value** |
| All cause | 379,848 | 9,362 | 1.00 (Ref.) | 10,751 | **1.05 (1.02; 1.08)** | **0.001** | 11,443 | **1.08 (1.05; 1.11)** | **<0.001** | 31,556 | **1.04 (1.02; 1.05)** | **<0.001** |
| Brain | 385,388 | 204 | 1.00 (Ref.) | 192 | 0.84 (0.69; 1.02) | 0.086 | 205 | 0.85 (0.69; 1.04) | 0.109 | 601 | 0.92 (0.83; 1.02) | 0.115 |
| Head & neck | 385,371 | 207 | 1.00 (Ref.) | 232 | 1.05 (0.87; 1.27) | 0.599 | 216 | 0.92 (0.75; 1.12) | 0.391 | 655 | 0.96 (0.87; 1.05) | 0.375 |
| Oesophagus | 385,375 | 184 | 1.00 (Ref.) | 273 | 1.19 (0.99; 1.44) | 0.064 | 336 | **1.29 (1.07; 1.55)** | **0.007** | 793 | **1.13 (1.03; 1.24)** | **0.008** |
| Lung | 385,114 | 679 | 1.00 (Ref.) | 933 | **1.15 (1.04; 1.27)** | **0.007** | 1,178 | **1.28 (1.16; 1.41)** | **<0.001** | 2,790 | **1.13 (1.08; 1.19)** | **<0.001** |
| Liver | 385,425 | 126 | 1.00 (Ref.) | 160 | 1.01 (0.80; 1.28) | 0.930 | 243 | **1.39 (1.11; 1.73)** | **0.004** | 529 | **1.20 (1.07; 1.34)** | **0.001** |
| Stomach | 385,407 | 131 | 1.00 (Ref.) | 173 | 1.10 (0.88; 1.38) | 0.410 | 205 | 1.17 (0.94; 1.47) | 0.161 | 509 | 1.08 (0.97; 1.21) | 0.163 |
| Pancreas | 385,375 | 261 | 1.00 (Ref.) | 355 | **1.19 (1.02; 1.40)** | **0.031** | 388 | **1.21 (1.03; 1.42)** | **0.021** | 1,004 | **1.09 (1.01; 1.18)** | **0.027** |
| Gallbladder | 385,452 | 42 | 1.00 (Ref.) | 80 | **1.63 (1.12; 2.37)** | **0.011** | 100 | **1.83 (1.27; 2.66)** | **0.001** | 222 | **1.32 (1.11; 1.57)** | **0.002** |
| Bladder | 385,342 | 191 | 1.00 (Ref.) | 236 | 1.03 (0.85; 1.25) | 0.762 | 289 | 1.15 (0.96; 1.39) | 0.138 | 716 | 1.08 (0.98; 1.18) | 0.122 |
| Kidney | 385,339 | 233 | 1.00 (Ref.) | 346 | **1.27 (1.08; 1.51)** | **0.004** | 432 | **1.47 (1.25; 1.73)** | **<0.001** | 1,011 | **1.21 (1.11; 1.31)** | **<0.001** |
| Colorectal | 384,798 | 1,063 | 1.00 (Ref.) | 1,226 | 1.05 (0.97; 1.14) | 0.232 | 1,391 | **1.15 (1.06; 1.25)** | **0.001** | 3,680 | **1.07 (1.03; 1.12)** | **0.001** |
| Colon | 385,025 | 711 | 1.00 (Ref.) | 824 | 1.05 (0.95; 1.16) | 0.321 | 966 | **1.20 (1.08; 1.32)** | **<0.001** | 2,501 | **1.10 (1.04; 1.15)** | **<0.001** |
| Proximal | 385,281 | 358 | 1.00 (Ref.) | 440 | 1.10 (0.95; 1.26) | 0.202 | 527 | **1.28 (1.11; 1.47)** | **0.001** | 1,325 | **1.13 (1.06; 1.21)** | **<0.001** |
| Distal | 385,254 | 312 | 1.00 (Ref.) | 350 | 1.03 (0.88; 1.20) | 0.727 | 395 | 1.12 (0.96; 1.30) | 0.154 | 1,057 | 1.06 (0.98; 1.14) | 0.146 |
| Rectum | 385,235 | 414 | 1.00 (Ref.) | 495 | 1.09 (0.95; 1.24) | 0.203 | 510 | 1.07 (0.94; 1.22) | 0.317 | 1,419 | 1.03 (0.97; 1.10) | 0.340 |
| Thyroid | 385,421 | 82 | 1.00 (Ref.) | 85 | 1.07 (0.79; 1.46) | 0.648 | 105 | **1.37 (1.01; 1.85)** | **0.042** | 272 | **1.17 (1.01; 1.37)** | **0.039** |
| Lymphatic | 385,030 | 903 | 1.00 (Ref.) | 1,006 | 1.00 (0.91; 1.09) | 0.963 | 1,074 | 1.03 (0.94; 1.12) | 0.589 | 2,983 | 1.01 (0.97; 1.06) | 0.576 |
| Leukaemia | 385,349 | 259 | 1.00 (Ref.) | 304 | 1.04 (0.88; 1.22) | 0.682 | 350 | 1.14 (0.97; 1.35) | 0.116 | 913 | 1.07 (0.99; 1.16) | 0.108 |
| Multiple Myeloma | 385,403 | 198 | 1.00 (Ref.) | 218 | 0.98 (0.81; 1.19) | 0.857 | 223 | 0.98 (0.80; 1.19) | 0.810 | 639 | 0.99 (0.90; 1.09) | 0.813 |
| Melanoma | 385,153 | 612 | 1.00 (Ref.) | 603 | 0.94 (0.84; 1.06) | 0.323 | 627 | 0.99 (0.89; 1.12) | 0.923 | 1,842 | 1.00 (0.94; 1.06) | 0.933 |
| Non-Hodgkin | 385,249 | 435 | 1.00 (Ref.) | 472 | 0.98 (0.86; 1.12) | 0.811 | 498 | 1.00 (0.88; 1.15) | 0.961 | 1,405 | 1.00 (0.94; 1.07) | 0.950 |
| Hodgkin | 385,458 | 28 | 1.00 (Ref.) | 30 | 0.89 (0.53; 1.50) | 0.660 | 33 | 0.84 (0.50; 1.42) | 0.523 | 91 | 0.92 (0.71; 1.19) | 0.529 |
| Prostate | 178,801 | 2,095 | 1.00 (Ref.) | 2,466 | 1.00 (0.94; 1.06) | 0.901 | 2,500 | **0.93 (0.87; 0.98)** | **0.014** | 7,061 | **0.96 (0.93; 0.99)** | **0.011** |
| Testis | 179,975 | 14 | 1.00 (Ref.) | 23 | 1.46 (0.75; 2.85) | 0.268 | 22 | 1.19 (0.60; 2.35) | 0.625 | 59 | 1.06 (0.77; 1.47) | 0.707 |
| Breast | 204,270 | 1,955 | 1.00 (Ref.) | 1,923 | **1.09 (1.02; 1.16)** | **0.007** | 1,689 | **1.11 (1.04; 1.19)** | **0.003** | 5,567 | **1.05 (1.02; 1.09)** | **0.002** |
| Breast Postmenopausal | 121,990 | 1,128 | 1.00 (Ref.) | 1,257 | **1.12 (1.03; 1.22)** | **0.006** | 1,120 | **1.13 (1.03; 1.23)** | **0.006** | 3,505 | **1.06 (1.02; 1.11)** | **0.006** |
| Breast Premenopausal | 51,012 | 587 | 1.00 (Ref.) | 380 | 1.03 (0.90; 1.17) | 0.652 | 236 | 1.01 (0.86; 1.18) | 0.948 | 1,203 | 1.01 (0.93; 1.09) | 0.860 |
| Uterine | 205,303 | 214 | 1.00 (Ref.) | 322 | **1.54 (1.30; 1.84)** | **<0.001** | 403 | **2.06 (1.73; 2.45)** | **<0.001** | 939 | **1.42 (1.31; 1.55)** | **<0.001** |
| Ovary | 205,353 | 222 | 1.00 (Ref.) | 216 | 1.01 (0.83; 1.22) | 0.949 | 233 | 1.17 (0.97; 1.42) | 0.102 | 671 | 1.08 (0.98; 1.19) | 0.103 |
| Cervix | 205,461 | 29 | 1.00 (Ref.) | 25 | 0.94 (0.55; 1.62) | 0.830 | 15 | 0.62 (0.33; 1.20) | 0.157 | 69 | 0.81 (0.59; 1.10) | 0.174 |

Data are presented in hazard ratio with 95% confidence intervals. The reference group was those in the lowest tertile. Model 1 was adjusted for age, income, deprivation, and ethnicity, Model 2 was adjusted by model 1 plus diet (red & process meat, fruits & vegetables, oily fish & alcohol), smoking, sedentary behaviour and physical activity and comorbidity.

**Table S4:** Association Between VAI Trends and Specific Cancer Sites Adjusted for BMI

| Cancer | HR 95% CI | P value |
| --- | --- | --- |
| All cause | 1.01 (0.99; 1.02) | 0.355 |
| Brain | 0.93 (0.83; 1.04) | 0.189 |
| Head & neck | 0.96 (0.87; 1.06) | 0.437 |
| Oesophagus | 1.02 (0.93; 1.12) | 0.721 |
| Lung | 1.10 (1.05; 1.16) | **<0.001** |
| Liver | 1.08 (0.96; 1.21) | 0.210 |
| Stomach | 0.99 (0.88; 1.11) | 0.834 |
| Pancreas | 1.02 (0.94; 1.11) | 0.655 |
| Gallbladder | 1.22 (1.02; 1.47) | **0.029** |
| Bladder | 1.01 (0.92; 1.12) | 0.824 |
| Kidney | 1.09 (1.00; 1.19) | **0.039** |
| Colorectal | 1.04 (0.99; 1.08) | 0.085 |
| Colon | 1.05 (1.00; 1.11) | 0.051 |
| Proximal | 1.09 (1.01; 1.17) | **0.022** |
| Distal | 1.01 (0.93; 1.09) | 0.831 |
| Rectum | 1.01 (0.94; 1.08) | 0.821 |
| Thyroid | 1.11 (0.94; 1.31) | 0.203 |
| Lymphatic | 0.99 (0.94; 1.04) | 0.634 |
| Leukaemia | 1.04 (0.95; 1.13) | 0.436 |
| Multiple Myeloma | 0.96 (0.87; 1.07) | 0.443 |
| Melanoma | 0.99 (0.93; 1.05) | 0.656 |
| Non-Hodgkin | 0.99 (0.92; 1.06) | 0.821 |
| Hodgkin | 0.84 (0.64; 1.11) | 0.223 |
| Prostate | 0.98 (0.95; 1.02) | 0.310 |
| Testis | 1.08 (0.77; 1.52) | 0.652 |
| Breast | 1.02 (0.99; 1.06) | 0.196 |
| Breast Postmenopausal | 1.02 (0.98; 1.07) | 0.367 |
| Breast Premenopausal | 1.04 (0.95; 1.13) | 0.395 |
| Uterine | 1.17 (1.08; 1.28) | **<0.001** |
| Ovary | 1.05 (0.95; 1.17) | 0.312 |
| Cervix | 0.75 (0.54; 1.04) | 0.086 |

Data are presented in hazard ratio with 95% confidence intervals per tertile increment on VAI. The reference group was those in the lowest tertile. Model was adjusted for age, income, deprivation, and ethnicity, diet (red & process meat, fruits & vegetables, oily fish & alcohol), smoking, sedentary behaviour and physical activity, multimorbidity count, and body mass index.
